# Nonalcoholic Fatty Liver Disease: Pathogenesis and Therapeutics from a Mitochondria-Centric Perspective

**DOI:** 10.1155/2014/637027

**Published:** 2014-10-13

**Authors:** Aaron M. Gusdon, Ke-xiu Song, Shen Qu

**Affiliations:** ^1^Department of Endocrinology and Metabolism, Shanghai 10th People's Hospital, School of Medicine, Tongji University, No. 301 Middle Yanchang Road, Shanghai 200072, China; ^2^Department of Neurology, Weill Cornell Medical College, New York, NY 10065, USA; ^3^Department of Endocrinology and Metabolism, Nanjing Medical University, Nanjing, Jiangsu 210029, China

## Abstract

Nonalcoholic fatty liver disease (NAFLD) describes a spectrum of disorders characterized by the accumulation of triglycerides within the liver. The global prevalence of NAFLD has been increasing as the obesity epidemic shows no sign of relenting. Mitochondria play a central role in hepatic lipid metabolism and also are affected by upstream signaling pathways involved in hepatic metabolism. This review will focus on the role of mitochondria in the pathophysiology of NAFLD and touch on some of the therapeutic approaches targeting mitochondria as well as metabolically important signaling pathways. Mitochondria are able to adapt to lipid accumulation in hepatocytes by increasing rates of beta-oxidation; however increased substrate delivery to the mitochondrial electron transport chain (ETC) leads to increased reactive oxygen species (ROS) production and eventually ETC dysfunction. Decreased ETC function combined with increased rates of fatty acid beta-oxidation leads to the accumulation of incomplete products of beta-oxidation, which combined with increased levels of ROS contribute to insulin resistance. Several related signaling pathways, nuclear receptors, and transcription factors also regulate hepatic lipid metabolism, many of which are redox sensitive and regulated by ROS.

## 1. Introduction

NAFLD is a broad term used to encompass a range of disorders ranging in severity from excess triglyceride accumulation in the liver to hepatic steatosis and eventually fibrosis, cirrhosis, and hepatocellular carcinoma. With the increasing prevalence of obesity and the metabolic syndrome, the prevalence of NAFLD has been reported to be about 20% [1]. Hepatic lipid accumulation results from a combination of uptake from circulating free fatty acids (FFAs),* de novo *lipogenesis, and dietary fats [2]. This lipid accumulation leads to hepatic steatosis, which is characterized by the accumulation of triglycerides (TGs) as lipid droplets within hepatocytes [3]. Progression to nonalcoholic steatohepatitis (NASH) is defined by steatosis with hepatocyte injury and the presence of inflammation, cell death, and fibrosis. Once a patient has developed nonalcoholic steatohepatitis (NASH), progression to cirrhosis can occur with about 10–29% of NASH patients developing cirrhosis within 10 years [4]. As with other causes of cirrhosis, those with cirrhosis secondary to NASH are at an increased risk of hepatocellular carcinoma, with 4–27% of patients with NASH induced cirrhosis developing hepatocellular carcinoma. It is not known whether steatosis also precedes NASH; however simple steatosis has often been observed to precede steatohepatitis. A number of factors contribute to the deposition of fat within the liver. Carbohydrate intake increases serum insulin levels which promotes lipogenesis. Furthermore, increased carbohydrate metabolism leads to increased abundance of acetyl-coenzyme A (acetyl-CoA), which can be used as a substrate for lipogenesis. Both insulin and glucose activate signaling pathways leading to lipogenesis. Insulin stimulates sterol regulatory element binding protein-1c (SREBP-1c), a transcription factor which targets many of the enzymes required in the lipogenesis pathway [5]. Glucose activates carbohydrate responsive element binding protein (ChREBP), another transcription factor upregulating the machinery for fatty acid synthesis [6].

While the factors leading from fat accumulation to the inflammatory changes and cell injury which occur in NASH have not yet fully been elucidated, a “two-hit” hypothesis has been suggested [7, 8]. The first hit has been suggested to be triglyceride accumulation within the hepatocyte. The second hit constitutes factors that increase oxidative stress and increase inflammation [7, 9, 10]. Mitochondria are at the center of cellular metabolism and not only generate energy from and provide energy for a variety of metabolic process but also are central for the coordination and integration of the various interwoven pathways defining the cell's metabolic program. This review will focus on alterations in mitochondrial function as well as the upstream signaling pathways which converge on the mitochondria and their role in the pathogenesis of NAFLD and implications for treatment.

## 2. The Role of Mitochondria in the Etiology of NAFLD

Excess of hepatic lipids lies at the heart of NAFLD, and mitochondria orchestrate hepatic lipid metabolism. FFAs can either enter the mitochondria to undergo beta-oxidation or undergo esterification into TGs. These TGs can then lead to the formation of lipid droplets in the liver or be secreted as very low density lipoproteins (VLDLs).

### 2.1. Mitochondrial Adaptations to NAFLD

Mitochondria play a pivotal role in fatty acid metabolism. Long chain fatty acids enter into the mitochondria utilizing carnitine palmitoyltransferase-1 (CPT-1) to undergo fatty acid oxidation and supply reducing equivalents for the mitochondrial electron transport chain (ETC). Typically, hepatocyte lipogenesis and lipid uptake are matched relatively equally with fatty acid oxidation and lipid export from the liver as cholesterol. Insulin resistance results in lipolysis in adipose tissue, increased concentration of fatty acids in the serum, and subsequently increased uptake by the liver. In an effort to compensate for increased fat deposition, liver mitochondria are able to increase the rate of fatty acid oxidation [11–16]. In ob/ob mice, increased rates of fatty acid oxidation were found to correlate with increased expression of the requisite enzymes [17–19]. While there is some disagreement in the literature, the majority of studies in humans suggest that increased fatty acid oxidation persists starting with simply fatty liver disease and continuing to mild and severe NASH [20–24]. The exact mechanism underlying the increase in fatty acid oxidation is imprecisely understood; however increased availability of the pool of free fatty acids to be used as substrates may be partially responsible [25, 26]. The involvement of other upstream signaling pathways will be discussed in depth later. While fatty acid oxidation is able to be upregulated to compensate for increased deposition of fat, multiple studies have shown that liver ATP levels are decreased in NAFLD [27–30]. Furthermore, ATP levels appear to decrease in parallel with the progression of NAFLD [28, 31]. This apparent discrepancy can likely be explained by changes in the enzymatic activities of the ETC. Increased expression of uncoupling protein 2 (UCP-2) expression resulting in uncoupling of ETC from ATP production may also contribute to decreased ATP levels [32, 33]. While a clear picture of the time course has not emerged, reductions in the enzymatic activity of the ETC complexes have been demonstrated in human and rodent models of NAFLD, with reductions in the activities of complex I [29, 34–37] and complex IV [17, 35–37] having been most often reported. Decreased ETC activity can lead to an overall impairment in mitochondrial respiration [38–41]; however discrepancies exist in the literature and may in part be due to a degree of uncoupling and thus increased respiration secondary to such factors as increased UCP-2 expression.

### 2.2. Mechanisms Leading to Mitochondrial Dysfunction

While metabolic adaptations occur to compensate for the increased liver fat load, mitochondrial dysfunction eventually occurs. Indeed, studies in human and rodents have demonstrated that enzymatic activities of the ETC complexes are reduced in NAFLD. While the exact mechanisms remain poorly understood, several processes have been proposed to contribute to impaired enzymatic activity. Increased generation of reactive oxygen species (ROS) has been demonstrated in NAFLD and likely plays a role in impairing ETC activity. Increased ROS production in NAFLD has been suggested to result from a more reduced quinone pool and an overall more reduced redox state within the mitochondrial matrix [42]. ROS have been documented to damage the ETC [43, 44] as well as causing mutants in the mitochondria DNA [45]. The ETC has also been shown to be sensitive to redox modulation by reactive nitrogen species (RNS) [46–48]. A study using ob/ob mice demonstrated tyrosine nitration of mitochondrial proteins and showed that ETC activity could be restored by scavenging reactive nitrogen species [17]. Increased TNF-*α* may contribute to RNS by increasing the expression of iNOS and resulting in peroxynitrite formation [17]. Indeed, TNF-*α* levels have been shown to be increased in NAFLD [49–51] and correlate with oxidative damage to mtDNA [52], and treating ob/ob mice with an anti-TNF antibody was shown to reverse the impaired ETC enzymatic activity in this model [17]. Decreased levels of adiponectin likely also contribute, although perhaps indirectly, to decreased ETC activity in NAFLD. Lower levels of adiponectin have been demonstrated in NAFLD [53–55], and adiponectin KO mice had decreased ETC enzymatic activities which were restored by adenovirus mediated expression of adiponectin [56]. Undoubtedly, the mechanisms leading to mitochondrial dysfunction in NAFLD are complex and multifactorial. Alterations of the related upstream signaling pathways alter in NAFLD and how they affect mitochondrial function will be addressed in more detail below.

### 2.3. Contribution of Oxidative Stress to the Pathogenesis of NAFLD

Early in the course of NAFLD, increased flow of reducing equivalents through the ETC provided from the increased beta-oxidation of fatty acids results in increased mitochondrial reactive oxygen species (ROS) production, which are derived primarily from complexes I and III [29, 32, 57, 58]. ROS is a blanket term used to refer to a variety of free radical species, and the primary form of ROS produced by the mitochondria is superoxide. Superoxide is generated in the mitochondria through the one electron reduction of oxygen at several sites within the ETC where a two-electron carrier donates electrons to a one electron carrier [59]. Sites within both complexes I and III have redox potentials making the generation of superoxide thermodynamically favorable. Within complex I, electron transfer from FMNH_2_ to Fe-S is thought to be the major site of superoxide generation while in complex III the transfer of electrons from ubiquinol to cytochrome *b*_*L*_ results in the formation of a ubisemiquinone radical capable of donating an electron to oxygen [60, 61].

Different types of lipids vary in their ability to lead to increased ROS production. Per molecule, polyunsaturated fatty acids provide more reducing equivalents to the ETC resulting in the production of more ROS and can inhibit glycolysis thereby shifting cellular metabolism away from glucose toward lipid utilization [62]. Polyunsaturated fatty acids can actually improve hepatic steatosis and reduce oxidative stress [63]. Lipids are not the only molecules contributing to oxidative stress in NAFLD. Free cholesterol has been shown to accumulate in the liver [64, 65] due to increased synthesis [66] and impairs hepatocyte antioxidant defenses by depleting mitochondrial glutathione [67]. This increased cholesterol burden also results in susceptibility to cytokine induced apoptosis [67].

However, the ETC is not the only source of ROS. As noted above, the ETC becomes progressively impaired in NAFLD, leading to an accumulation of FFAs in the cytosol that cannot be completely oxidized. These FFAs can be oxidized by peroxisomal beta-oxidation or microsomal omega oxidation. Peroxisomal fatty acid oxidation leads to hydrogen peroxide production [68], while microsomal fatty acid oxidation leads to oxidative stress through the ability of cytochrome P4502E1 and cytochrome P4504A to partially reduce oxygen [69]. Indeed, in models of steatosis, peroxisomal and microsomal fatty acid oxidation has been shown to be increased [20, 70].

ROS can react with fatty acids leading to lipid peroxidation and the formation of reactive aldehydes such as trans-4-hydroxy-2-nonenal (4-HNE) and malondialdehyde (MDA) [71]. Oxidatively modified proteins have been shown to accumulate in NAFLD [72, 73], and peroxidation of mitochondrial membrane phospholipids may further contribute to ETC decline [74]. Interestingly, 4-HNE has been shown to form adducts with UCP-2 leading to an increase in its activity [32] perhaps explaining some of the uncoupling and decreased ATP production in NAFLD.

Recent data have suggested that oxidative damage to cardiolipin may play a role in impaired insulin signaling and the metabolic syndrome. ALCAT1 catalyzes the synthesis of a form of cardiolipin that is more oxidatively sensitive and itself may also be upregulated by oxidative stress. ALCAT1 knockout was further shown to preserve mitochondrial function, prevent diet induced obesity, and improve signaling indicating that the oxidative modification of cardiolipin may be an important mechanism linking oxidative stress with insulin resistance and NAFLD [75].

Increased ROS production also results in increased expression of a number of cytokines which have been shown to play a pathological role in NAFLD including Fas ligand, TNF-*α*, TGF-beta, and IL-8 [76]. TNF-*α* and IL-6 have both been found to be elevated in the liver and serum of patients with steatohepatitis [77], and normalization of the levels of these cytokines has been shown to blunt the progression of NAFLD [78].

While mitochondrial dysfunction plays a key role in NAFLD as well as insulin resistance, it has been unclear at exactly which stage in the pathogenesis of these conditions mitochondrial function becomes altered. One recent study has suggested that insulin resistance precedes overt mitochondrial dysfunction [79]. However, this study did not quantify the time course of increased reactive oxygen species production but did demonstrate that oxidative stress was critical for the inevitable decline in mitochondrial function [79]. Therefore, it is conceivable that increased ROS production may well precede impaired insulin signaling and mitochondrial dysfunction, which will be discussed in more depth later.

### 2.4. Antioxidant Based Therapies

Given the abundance of data suggesting that oxidative stress plays a key role in the progression of NAFLD, several studies have assessed the efficacy of antioxidant based therapies. Vitamin E is an antioxidant with the ability to prevent the propagation of free radicals and has therefore been a logical choice in the treatment of NAFLD [80]. A small pilot study of 16 patients with biopsy proven NASH demonstrated increased levels of proinflammatory cytokines in the NASH patients [81]. The study was conducted over a 12-week period with lifestyle modification resulting in an improvement in liver function tests (LFTs); however there was no independent effect of vitamin E treatment [81]. Longer treatment duration of 6 months with vitamin E and vitamin C in 45 patients did demonstrate a statistically significant improvement in hepatic fibrosis after vitamin treatment; however there was significant variability among the patients and no effect on the level of inflammation [82]. Similar treatment duration of vitamin E in children with NASH demonstrated improvement in LFTs [83]. More recently, the PIVENS trial performed over two years with vitamin E or pioglitazone in nondiabetic patients with NALD showed improvement in steatohepatitis on posttreatment liver biopsies [84]. While there may be a benefit of vitamin E treatment for NAFLD, enthusiasm has been tempered by a meta-analysis showing increased all-cause mortality from high dose vitamin E treatment [85], although some have questioned these results [86]. Given the available data, vitamin E therapy is only recommended in nondiabetic NAFLD patients with biopsy proven NASH [87]. While vitamin E has been the most studied antioxidant compound in NASH, various others have been studied. Betaine is an antioxidant and choline derivative which decreases oxidative stress by increasing levels of s-adenosyl-methionine through enhanced homocysteine recycling [88]. A study treating rats with diet induced NAFLD with betaine found decreased liver fat accumulation, decreased TNF-*α* levels, and increased cytosolic antioxidants [51]. However, clinical trials with betaine have not been as promising. In a study of 35 patients with biopsy proven NASH, while 12 months of betaine treatment slowed the progression of steatosis, it has no effect on serum insulin and glucose or proinflammatory cytokines and antioxidants [89]. N-Acetylcysteine (NAC) is glutathione precursor capable of detoxifying nitric oxide as well as blocking lipid peroxidation and thus was another logical choice. A study in Wistar rats suggested that S-nitroso-N-acetylcysteine (SNAC) was able to prevent the development of NAFLD induced by a choline deficient diet. Another study confirmed that SNAC treatment was able to prevent the development of microvascular steatosis in ob/ob mice and went on to demonstrate that it could reverse microvascular steatosis [90]. A study in Sprague-Dawley rats showed that the protective effects of SNAC may be due to its ability to inhibit the progression of fibrosis, as SNAC treatment decreased liver area occupied by collagen and resulted in downregulation of TGF-*β*1, HSP-60, collagen-1*α*, and tissue inhibitors of metalloproteinase-2 [91]. Studies in other models of cirrhosis have confirmed the antifibrotic effects of SNAC [92, 93]. However, large scale clinical trials have yet to be conducted, and initial trials have not been overly encouraging. 35 patients with biopsy proven NASH included in a study of were treated with NAC or placebo for 4 weeks without a significant benefit of NAC on LFTs [94]; however the study did not include a posttreatment biopsy to assess the extent of fibrosis. Another more recent study of 30 patients with NALD treated with NAC versus vitamin C demonstrated an improvement in LFTs with NAC treatment but was limited by its lack of placebo control group and also had no posttreatment biopsy [95]. Resveratrol, which has been implicated in improved cardiovascular outcomes and is the agent supposedly responsible for the “French paradox” [96], has also been studied in NAFLD. It has been shown to reduce hepatic lipogenesis [97], decrease oxidative stress [98], and improve steatosis [98, 99] in models of NAFLD perhaps through activation of Sirt1 [100, 101] and AMP activated kinase (AMPK) [102]. Resveratrol's ability to activate AMPK may be due to inhibition of mitochondrial complexes III and V therefore impairing ATP synthesis [103, 104]. However, data from clinical trials are lacking.

In additional to conventionally thought of antioxidants, part of the ability of metformin to improve insulin sensitivity and reduce hepatic steatosis may be due to decreased mitochondrial ROS production. In rats, metformin was shown to decrease reverse electron transport mediated ROS production at complex I in isolated mitochondria perhaps due to a reduction in mitochondrial membrane potential [105]. However, the ability of metformin to suppress ROS has not been demonstrated* in vivo*, and it is unclear to what extent reverse electron transport contributes to mitochondrial ROS production with physiological substrates [106]. Metformin also has a number of important signaling effects which will be discussed further later. Clinical trials studying metformin in patients with NASH have shown an improvement in aminotransferases while not having a significant effect on liver histology [107–109]. In a randomized control trial, Haukeland et al. also found no effect of 6 months of treatment with metformin on liver histology [110]. A recent meta-analysis failed to demonstrate a benefit of metformin treatment for NAFLD when looking at liver histology or aminotransferases [111]. Therefore, the current guidelines do not recommend metformin for the specific treatment of NASH [87].

## 3. Insulin Resistance

### 3.1. Mechanism of Insulin Resistance and Its Association with NAFLD

There is a clear association between insulin resistance and NAFLD, with impaired insulin signaling almost always occurring in conjunction with NAFLD. Given the close association between the two, each has been implicated in the pathogenesis of the other; however debate continues as to whether insulin resistance is a cause or consequence of hepatic steatosis and NAFLD. While insulin typically promotes glucose uptake as well as lipogenesis, insulin resistance describes resistance against the ability of insulin to trigger glucose uptake in liver, muscle, and adipose tissue. Insulin stimulates glycolysis primarily through activation of glucokinase [112]. Insulin also activates sterol regulatory element binding protein 1-c (SREBP-1c), a transcription factor which stimulates liver pyruvate kinase, acetyl-CoA carboxylase, fatty acid synthase, stearoyl-CoA desaturase, and Spot 14, all of which are involved in fatty acid synthesis [113]. Given that insulin acts to increase lipogenesis, it might be thought that, in conditions of insulin resistance, fatty acid synthesis would be decreased. However, it has been shown that, in states of insulin resistance, insulin loses its ability to decrease glucose production, while maintaining the ability to stimulate lipogenesis [114]. For instance, even with significant insulin resistance as determined by serum glucose levels, insulin was able to activate SREBP-1c and promote lipogenesis [115].

Studies in several animal models have suggested that insulin resistance precedes and is a cause of subsequent hepatic steatosis [116]. Hepatic steatosis was also shown to occur in human populations with postreceptor mutations in the insulin signaling pathway leading to insulin resistance [117]. However, while the lipogenic effects of insulin and the hyperinsulinemia that is characteristic of insulin resistance likely play a role in the accumulation of hepatic fat, it has long been suspected that hepatic lipids precipitate the development of insulin resistance. One early hypothesis proposed that an accumulation of hepatic lipids in combination with decreased mitochondrial beta-oxidation of fatty acids reroutes these fatty acids toward the production of diacylglycerol and ceramide [118–122] resulting in the induction of stress induced kinases and subsequently impaired insulin signaling [123, 124]. However, this hypothesis has fallen out of favor recently for a number of reasons. First of all, it has been shown that accumulation of DAG and ceramide does not obligatorily lead to insulin resistance and is unlikely to be causative in insulin resistance [125]. Furthermore, in mice with genetic defects impairing fatty acid mobilization or oxidation, hepatic steatosis occurred without insulin resistance [116, 126]. One study generated mice with liver overexpression of acyl-CoA:diacylglycerol acyltransferase 2 (DGAT2) and showed that while these mice developed hepatic steatosis, they did not develop glucose or insulin intolerance [127]. These studies provided support for the notion that steatosis can occur without preceding insulin resistance and also called into question the idea that hepatic lipid accumulation leads to insulin resistance. Additionally, increased hepatic fatty acid beta-oxidation using PPAR agonists did not improve insulin resistance in type 2 diabetic patients [128] and has actually been shown to worsen insulin resistance in some rodent models [128–130].

Recently, support has grown for the hypothesis that increased fatty acid beta-oxidation actually leads to insulin resistance. In a series of elegant experiments, Koves et al. demonstrated that the increased beta-oxidation that occurred in high-fat diet fed rats led to the accumulation of incompletely oxidized fatty acid intermediates and also depleted TCA cycle intermediates [131]. In their study, lipid induced insulin resistance was actually reversed by blocking fatty acid beta-oxidation. Furthermore, data are accumulating to suggest that an excess lipid burden renders cells less metabolically flexible, that is, less able to switch between fatty acid oxidation and glucose oxidation. Recent work has suggested that carnitine acetyltransferase (CrAT) may permit much of this flexibility. CrAT converts acetyl-CoA to its esterified membrane permeable form, thus allowing mitochondrial utilization of acetyl-CoA and relieving the feedback inhibition exerted by acetyl-CoA. The importance of CrAT and the metabolic flexibility that it affords was confirmed by mice harboring a muscle specific knockout which subsequently develop glucose intolerance [132].

The idea that excess oxidation of fatty acids may impair glucose uptake and catabolism is not new and was actually proposed in 1963 by Randle et al. in what has come to be known as the Randle cycle [133]. Certainly, it makes evolutionary sense that, during periods of increased fatty acids oxidation, the body would want to maintain serum glucose levels. Fatty acids are used as the preferred fuel source primarily during the fasted state, and given the paramount importance of maintaining serum glucose concentrations in states of nutrient depletion, mechanisms have evolved to inhibit glucose oxidation during fatty acid oxidation [134]. Indeed, fatty acid oxidation has been shown to inhibit several steps along the pathway of glucose utilization. Fatty acid oxidation exerts its greatest inhibitory effect on pyruvate dehydrogenase (PDH) activity. Fatty acid oxidation leads to an increase in the ration of NADH to NAD+ as well as acetyl-CoA to CoA, which results in feedback inhibition of PDH activity in part due to activation of pyruvate dehydrogenase kinase (PDK) [135]. This leads to an accumulation of citrate, which is an allosteric inhibitor of phosphofructokinase-1 (PFK-1) leading to an increase in glucose 6-phosphate, which inhibits hexokinase [136]. Inhibition of PDH also preserves intracellular levels of pyruvate and lactate to be used for gluconeogenesis [137]. Therefore, treatment strategies that have been devised to increase fatty acid oxidation may actually have a deleterious effect on insulin resistance, which may further exacerbate lipid accumulation and NAFLD.

These findings fit well with the mitochondrial adaptations that occur in NAFLD, which were discussed above. Increased mitochondrial beta-oxidation initially acts as a compensatory mechanism to deal with the increased deposition of FFAs in the liver. However, this increased fatty acid oxidation comes at a cost, primarily increased ROS production which occurs secondary to increased substrate supply without increased energy demand exacerbated in conditions of decreased exercise. These may also explain part of the beneficial effects of exercise on insulin resistance. Mitochondrial function remains intact early in this process as studies have demonstrated that insulin resistance clearly precedes the development of mitochondrial dysfunction [79].

Overproduction of ROS likely plays a key mechanistic role linking fatty acid oxidation with insulin resistance. In cultured hepatocytes, it has been shown that saturated fatty acids are able to induce increased mitochondrial ROS production, which resulted in c-Jun NH(2)-terminal kinase (JNK) activation and subsequently insulin resistance as demonstrated by decreased insulin stimulated tyrosine phosphorylation of IRS-2 and serine phosphorylation of Akt [138]. Inhibition of CPT-1 with etomoxir has also been shown to block the insulin resistance in cell lines induced by saturated fatty acid treatment [138, 139].

However, the role of ROS production in insulin resistance is likely more complex. At low dose, ROS can actually enhance insulin signaling by inhibition of protein-tyrosine phosphatase (PTP1B) [140, 141], which is known to negatively regulate insulin signaling [142]. Antioxidant supplementation has also been shown to attenuate the insulin sensitizing effects of physical exercise [143, 144], which is also known to increase mitochondrial ROS production [145]. Additionally, ROS likely serve as important signaling molecules upregulating PGC-1*α* expression and various antioxidant enzymes [145–147]. Thus studies using antioxidants in NAFLD should be interpreted in light of not only the deleterious effects of ROS but also their signaling roles.

Further complicating the use of antioxidant therapy in NAFLD is the specificity of ROS which are scavenged. For instance, scavenging superoxide with superoxide dismutase (SOD) produces hydrogen peroxide and has been shown to actually exacerbate steatohepatitis unless glutathione, which scavenges hydrogen peroxide, was replenished [148].

### 3.2. Improving Insulin Signaling through the Reduction of Hepatic Fatty Acid Oxidation

The accumulation of hepatic lipids in NAFLD had initially provided the impetus to devise therapeutic approaches to increase hepatic lipid metabolism. Some initial success with decreasing hepatic lipids was found with compounds such as 3,5-diiodo-l-thyronine [149]. Other approaches have aimed at increasing CPT-1, the rate limiting step in fatty acid beta-oxidation [150]. While lipid accumulation in the liver causes NAFLD, the process of fatty acid oxidation also contributes to the pathogenesis of NAFLD, as discussed above. Indeed, Koves et al. demonstrated that reducing rates of fatty acid beta-oxidation by knocking down malonyl-coA decarboxylase imparted resistance against diet induced glucose intolerance [131]. Pharmacological approaches to inhibiting CPT-1 have also been developed. Etomoxir (ethyl-2-[6-(4-chlorophenoxy) hexyl]-oxirane-2-carboxylate) blocks CPT-1 and its use in humans has been shown to decrease plasma glucose levels and was accompanied by increased expression of GLUT4 on the cell membranes of myocytes [151]. In mice treated with etomoxir, insulin signaling was improved even though diacylglycerol and triacylglycerol were shown to accumulate within myocytes [151]. A recent study treated diet induced obese mice with the CPT-1 inhibitor oxfenicine and found improved glucose tolerance and insulin sensitivity as well as evidence of improved insulin signaling and decreased toxic metabolites of lipid metabolism [152]. Similarly, the CPT-1 inhibitor teglicar has been shown to decrease endogenous glucose production as well as improve insulin sensitivity in rat and mice fed a high-fat diet [153].

## 4. Signaling Pathways and Nuclear Receptors

**Figure 1:**
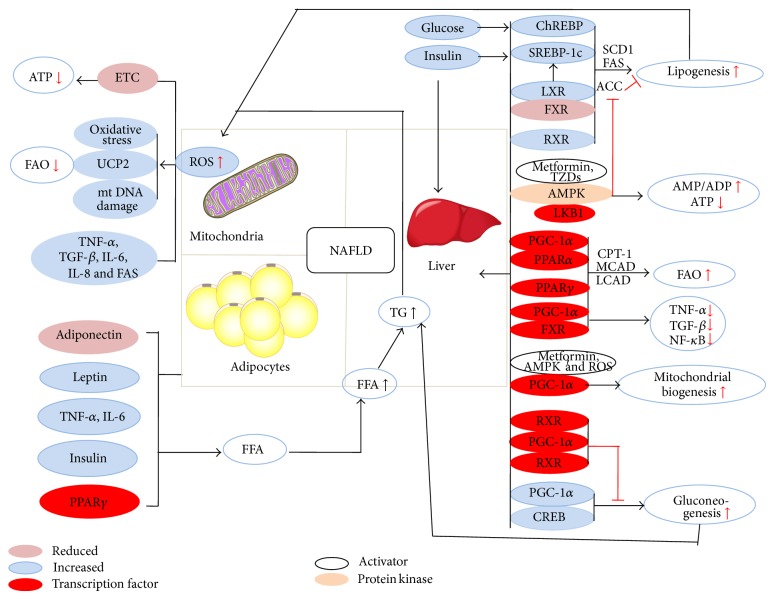
Pathways involved in the development of NAFLD. The role of mitochondria is highlighted as increased reactive oxygen species (ROS) production leads to inactivation of the ETC resulting in decreased ATP levels and impaired energy supply. Mitochondrial damage by ROS also leads to impaired fatty acid oxidation (FAO). Incomplete FAO may also occur as a result of ROS production and contribute to insulin resistance and further hepatic fatty acid accumulation. ROS production also leads to the activation of a number of cytokines known to play a role in the pathogenesis of NAFLD. Insulin signaling plays a key role in NAFLD. Insulin signals through SREP-1c, which activates lipogenesis. In states of insulin resistance, this ability to stimulate lipogenesis is maintained while the ability to stimulate glucose uptake is reduced. Glucose stimulates lipogenesis as well but does so by activating ChREBP. LXR/FXR and RXR also play key roles in increasing lipogenesis. Signaling through AMPK decreases lipogenesis by inhibiting ACC. However, AMPK also has very broad effects on the cellular energy state leading to decreased levels of ATP and subsequent activation of pyruvate kinase, phosphofructokinase, and pyruvate dehydrogenase. The PPAR family of transcription factors plays an important role in lipid metabolism with PPAR*α* stimulating fatty acid oxidation and PPAR*γ* increasing peripheral update of lipid by adipocytes. PGC-1*α* is a transcription factor which can lead to activation of gluconeogenesis when activated by CREB or ROS production. However PGC-1*α* also leads to increased mitochondrial biogenesis when activated by AMPK or metformin which may ameliorate NAFLD.

To this point, control of metabolism and its alterations in NAFLD have been discussed at the cytosolic and mitochondrial levels. However, the metabolic program of the cell is intricately controlled by a set of interwoven singling pathways, transcription factors, and nuclear receptors (Figure 1).

### 4.1. AMPK

As discussed above, mitochondria have the ability to adapt to the changing metabolic demands of the cell, and several elegant and interwoven pathways allow them to do so. Among the most important and well-studied factor is AMPK. AMPK is a serine/threonine protein kinase which acts as a sensor of the cell's energy state. An increased ratio of AMP to ATP indicates a relative energy deficiency and results in activation of AMPK [154]. AMPK is a heterotrimer with its alpha subunits performing catalytic functions and its beta and gamma subunits serving regulatory roles. Displacement of ATP bound to the regulatory domain by AMP or ADP results in allosteric activation of AMPK [155], protects the alpha subunit from dephosphorylation, and promotes the phosphorylation of threonine 172 causing additional activation [156]. Carbohydrates such as glycogen can inhibit AMPK by binding to a site within the beta subunit [157]. Upstream kinases also play a role in the activation of AMPK with liver kinase B1 (LKB1) and calcium calmodulin-dependent protein kinase *β* (CAMKK*β*) both catalyzing the activating phosphorylation of threonine 172, the former due to changes in energy state and the latter due to calcium influxes [158, 159]. Activation of AMPK turns on catabolic processes, which increase the availability of cellular ATP, such as fatty acid oxidation, and turns off processes like lipid synthesis which consume ATP [160, 161].

It has been suggested that the glucose lowering effect of AMPK activation results from its interaction with acetyl-coA carboxylases. Several studies have demonstrated the importance of acetyl-CoA carboxylases 1 and 2 (ACC1 and ACC2) in the development of hepatic steatosis and hepatic insulin resistance. These enzymes generate malonyl-CoA which not only serves as a precursor of fatty acid synthesis but also inhibits fatty acid oxidation by inhibiting CPT-1. Inhibition of both ACC1 and ACC2 decreases levels of malonyl-CoA, lowers hepatic lipids, and improves insulin sensitivity in a high-fat diet rat model of NAFLD [162]. ACC1 has been shown to be phosphorylated by AMPK [163, 164], inhibiting the synthesis of malonyl-CoA. In elegant experiments replacing the serine residues phosphorylated by AMPK with alanine, Fullerton et al. demonstrated that the inability to phosphorylate ACC1 leads to glucose intolerance and hepatic insulin resistance [165]. Importantly, their study demonstrated increased hepatic lipogenesis, glucose production, and insulin sensitivity in the fed state [165].

Activation of fatty acid oxidation may be detrimental for the metabolic syndrome given its reciprocal inhibition of glucose uptake and oxidation. Therefore, the ability of AMPK to stimulate fatty acid beta-oxidation might be expected to have some untoward effects on NAFLD and insulin resistance. However, it has been demonstrated that AMPK activation overcomes the inhibitory effect of fatty acid oxidation on glucose utilization [166, 167]. While AMPK promotes fatty acid oxidation by ACC inactivation and subsequently decreased levels of malonyl-CoA, it also stimulates glucose uptake and glycolysis. Indeed, AMPK has been shown to act in an insulin independent fashion to stimulate glucose uptake [160]. AMPK exerts its effects on lipid and carbohydrate metabolism by targeting a number of key enzymes [161]. AMPK inhibits ACC, which decreases the level of malonyl-CoA both decreasing lipogenesis and relieving the inhibition on fatty acid beta-oxidation exerted by malonyl-CoA on CPT-1 [168]. AMPK stimulates phosphofructokinase (PFK-2), which stimulates glycolysis [169]. AMPK also improves glucose transport and increases glycolysis by recruiting GLUT4 to the plasma membrane due to phosphorylation of Akt substrate 160 [170].

Interest in modulation of AMPK for treatment of metabolic conditions arouse in part due to the observation that it is able to stimulate glucose uptake in an insulin dependent fashion [171, 172]. AMPK has also been implicated in the action of biguanides and thiazolidinediones and will be discussed later [160].

In addition to activation of AMPK, simply decreasing the energetic state of the hepatocyte may have important effects on decreasing hepatic gluconeogenesis and decreasing serum glucose levels. Decreased cellular ATP levels relieve the feedback inhibition exerted by ATP on L-type pyruvate kinase [173] and phosphofructokinase [173] as well as pyruvate dehydrogenase [174]. AMP also can increase the ability of 2,6-bisphosphate to stimulate phosphofructokinase and inhibit fructose-1,6-bisphosphatase [175].

Given its ability to increase pathways leading to lipid catabolism, activation of AMPK has been targeted in therapeutic approaches for NAFLD. Interestingly, some of the beneficial effects of metformin may be due to its ability to modulate AMPK signaling. Metformin has long been known to improve insulin sensitivity [176–178] and decrease hepatic gluconeogenesis, and it has therefore logically been investigated as a treatment option for NAFLD. Initial interest for this concept came from a study showing that metformin can activate AMPK in liver and skeletal muscle and that inhibition of AMPK blocked the ability of metformin to decrease hepatocyte glucose production [179]. However, it has been suggested that metformin can also act independently of AMPK, with AMPK knockout mice still demonstrating decreased gluconeogenesis and lower blood glucose levels after metformin treatment [180]. This study went further to show that metformin decreased cellular levels of ATP and increased the ratio of AMP to ATP [180]. These results are not unexpected in light of data indicating that metformin can inhibit complex I of the mitochondrial ETC [181, 182]. While perhaps not exclusively inhibiting complex I [183] and likely having broader less specific effects by binding to membrane phospholipids [184], it is clear that metformin can alter the overall energetic state of the cell. Increased AMP levels induced by metformin treatment have also been shown to inhibit adenylate cyclase thereby reducing levels of cAMP and consequently PKA activation [185]. Impaired ability to activate PKA inhibits the downstream signaling effects of glucagon and reduces fasting glucose levels [185]. Therefore inhibition of adenylate cyclase via increased AMP levels likely represents an important mechanism by which metformin reduces blood glucose levels. Thus, whether it is through activation of AMPK or otherwise, much of the effect of the beneficial effect of metformin is likely derived from modulation of cellular energy balance [186].

In addition to metformin, small molecules have been designed to specifically target AMPK. The small molecule A-769662, a member of a thienopyridone family of AMPK activators, was shown to increase fatty acid oxidation, downregulate the machinery necessary for lipogenesis, and reduce plasma triglyceride and glucose levels [187]. Cardiotrophin is a cytokine with known hepatoprotective effects and has been shown to decrease hepatic triglyceride accumulation and reduce inflammation in a model of NAFLD largely through its ability to activate AMPK [188]. 5-Aminoimidazole-4-carboxamide-1-beta-D-ribofuranoside (AICAR) is another pharmacological activator of AMPK, which is metabolized to ZMP, an analog of AMP. It has been shown to have beneficial effects on insulin sensitivity and improves glucose uptake in models of type 2 diabetes and may also be of benefit in NAFLD [189, 190]. Alpha-lipoic acid is an antioxidant which has been shown to activate AMPK and decrease hepatic steatosis [191]. Other AMPK activating compounds have been developed as well; however clinical trials are still pending for most of these drugs.

### 4.2. Peroxisome Proliferator-Activated Receptors (PPARs)

Three different PPAR isoforms exist: *α*, *γ*, and *β*/*δ* [192]. PPAR*α* has an important role in increasing fatty acid oxidation in mitochondria, peroxisomes, and microsomes [193–195]. PPAR*α* forms a heterodimer with retinoid-X-receptor (RXR) which then binds to its nuclear response element [196]. PPAR*α* is thought to act as a sensor of hepatic lipid balance, and fatty acids have been shown to bind to PPAR*α* leading to its activation and thus increased beta-oxidation [197]. Some studies have suggested that deficiency of PPA*α* contributes to hepatic steatosis [198]. Fibrates are the most important class of drugs known to act as PPAR*α* agonists and have been used as treatment options for diabetes and the metabolic syndrome. A number of animal studies have suggested that PPAR*α* agonists may be of benefit for NAFLD [51, 199, 200]. However, only a few clinical studies have been conducted and demonstrated minimal benefit. After a 54-week treatment course with either fenofibrate or atorvastatin or both, a study of 186 patients with liver steatosis showed no biochemical or imaging evidence of NAFLD in 42% of patients treated with fenofibrate compared with 67% treated with atorvastatin and 70% treated with both [201]. Again, the beneficial effects observed in this study may have been due to the effect of fenofibrate on lowering TG and LDL levels [201]. In another small study of 16 patients with biopsy proven NAFLD, triglyceride levels were again improved and insulin resistance was moderately improved; however there was not histological improvement on repeated liver biopsy [202].

As discussed above, unopposed activation of fatty acid beta-oxidation may lead to reduced glucose uptake and utilization via the Randle cycle. Indeed, overexpression of PPAR*α* has been shown to be detrimental to cardiomyocytes resulting in impaired glucose and cardiomyopathy reminiscent of that seen in patients with uncontrolled diabetes [203]. Therefore, targeting PPAR*α* may not be the optimal strategy for treating NAFLD. Nevertheless, the fibrate class of drugs agonizes PPAR*α* and several studies have shown a beneficial effect. This benefit may be due to PPAR*α*'s broader effect on lipid metabolism. Indeed, PPAR*α* has been shown to decrease plasma triglyceride levels largely through its ability to increase lipoprotein lipase mediated triglyceride clearance as well as decreasing the availability of triglycerides for VLDL secretion [204]. Furthermore, PPAR*α* has been shown to lower TNF-*α* levels [205], another effect that could be beneficial in NAFLD.

PPAR*γ* is targeted by the thiazolidinediones (TZDs), which have been shown to be of benefit in type 2 diabetes. It has been shown to be critical for the uptake and storage of triglycerides by adipose tissues [206], thereby lowering serum triglyceride levels and reducing the amount of substrate available for hepatic fatty acid beta-oxidation. Compared with PPAR*α*, a larger number of clinical trials have been conducted looking at PPAR*γ* agonists, primarily TZDs, in the treatment of NAFLD. One early study demonstrated that treatment with rosiglitazone reduced steatosis and necroinflammation in patients with biopsy confirmed NASH [207]; however more recent studies have suggested that rosiglitazone had no effect on necroinflammation or fibrosis but recapitulated its ability to reduce hepatic steatosis [208, 209]. Other studies using pioglitazone have shown that this thiazolidinedione is able to improve hepatic steatosis and markers of inflammation as well as decrease hepatic fibrosis [210, 211]. Importantly, in the PIVENS trial, which compared pioglitazone or vitamin E treatment with placebo, while a pioglitazone treatment did not reach significance for reduction in NAFLD activity score, significantly more patients achieved resolution of NASH with pioglitazone than with control and histological benefits were demonstrated on liver biopsy [84]. While the guidelines suggest that pioglitazone can be used for treatment of biopsy proven NASH [87], its use has also been associated with a higher rate of congestive heart failure [212]; therefore it should be used with extreme caution and in patients who have cardiac comorbidities. Of note, pioglitazone has led to weight gain in a number of the studies mentioned, which is perhaps not unexpected given the effect of PPAR-gamma on increasing FFA incorporation into peripheral adipose tissue. TZDs have also been shown to activate AMPK [213], which may be partially due to their ability to alter the energy state of the cell [214, 215] but is likely also adiponectin [216, 217] and PPAR mediated [218].

PPAR *β*/*δ* targets genes involved in fatty acid oxidation, glucose metabolism, and mitochondrial respiration [219, 220]. A PPAR *α* and *δ* agonist, GFT505, has been shown to improve liver steatosis in a diet induced rodent model; however its effects may have been primarily due to the observed decrease in hepatic inflammation [221]. Similar results were also obtained with the PPAR pan-agonist bezafibrate, which may also play a significant anti-inflammatory role [222].

### 4.3. PGC-1*α*

PGC-1*α* is a key transcription factor which stimulates the expression of mitochondrial genes as well as nuclear genes required for mitochondrial biogenesis [223]. PGC-1*α* stimulates mitochondrial transcription factor A (TFAM) secondary to nuclear respiratory factors (NRFs) 1 and 2 activation [224]. In liver biopsies from NAFLD patients, one study demonstrated that PPARGC1A, the gene encoding PGC-1*α*, was epigenetically modified by methylation leading to decreased liver mitochondrial content [225]. This reduction in mitochondrial mass in combination with increased flux through mitochondrial beta-oxidation may further exacerbate the buildup of incompletely oxidized metabolites and further exacerbate insulin resistance. However, PGC-1*α* has also been shown to act downstream of cAMP response binding protein (CREB) to induce gluconeogenesis [226] in part due to its ability to activate phosphoenolpyruvate carboxykinase (PEPCK) and glucose-6-phosphatase [227]. Therefore, activation of PGC-1*α* may also contribute to insulin resistance and increased serum glucose levels. PGC-1*α* is known to act as a redox sensor and can be activated by oxidative stress [228, 229]. As discussed above, oxidative stress likely plays a key role in the development of insulin resistance. Kumashiro et al. confirmed the presence of increased liver ROS levels in obese diabetic *db*/*db* mice and showed that overexpression of superoxide dismutase 1 (SOD1) in the liver of these mice improved insulin sensitivity [230]. They went on to suggest that improved insulin sensitivity was due to decreased ROS induced expression of PGC-1*α* and the gluconeogenic genes it is known to induce. The ability of PGC-1*α* to increase the expression of mitochondrial genes while also increasing the expression of gluconeogenic genes may therefore seem to have contrasting effects on the pathogenesis of NAFLD. However, whether PGC-1*α* is beneficial or detrimental for NAFLD may depend on the context in which it is activated. As an example, as discussed before, AMPK has a number of beneficial effects in NAFLD, and it is also known to control the expression of PGC-1*α* by phosphorylation and deacetylation [231]. Still, while AMPK activates PGC-1*α* it does not increase gluconeogenesis. The explanation for this observation may be found in the effect that AMPK has on other transcription factors. The ability of CREB to induce gluconeogenesis depends on its ability to bind to coactivators such as CRTC2, which allows for the assembly of the transcriptional machinery [232]. AMPK phosphorylates CRTC2 leading to its export into the cytosol after binding to 14-3-3 protein [232, 233]. Transcriptional activity of HNF-4*α* also increases hepatic gluconeogenesis [234], and again AMPK has been shown to decrease its levels by both direct phosphorylation and impairing protein stability [235, 236]. PGC-1*α* activation secondary to AMPK activation may not be able to induce gluconeogenesis given the unavailability of the necessary coactivators. Similarly, metformin has also been shown to increase the expression of PGC-1*α* and stimulate transcription of mitochondrial genes, while not resulting in activation of gluconeogenesis [237]. While part of metformin's inhibitory effect on gluconeogenesis is likely secondary to activation of AMPK [238], change in the cell's energy state also has been shown to play a role independently of AMPK [180]. Therefore, the effects of targeting PGC-1*α* independently of AMPK will depend on the status of other transcription factors as well as the energy state of the cell and may lead to impaired glucose tolerance if gluconeogenesis is also upregulated.

### 4.4. Liver X Receptor (LXR)

LXRs are transcription factors that are critical for the control of lipid homeostasis and act by binding to LXR-response elements [239]. LXRs are also cholesterol sensors and regulate the absorption and metabolism of sterols. LXRs promote hepatic steatosis indirectly by activating SREBP1c [240] and have also been shown to directly regulate expression of genes involved in lipid synthesis such as fatty acid synthase [241]. Increased expression of LXR may be a key mediator of hepatic steatosis in NAFLD and has also been correlated with increased liver expression of SREBP-1c [242]. There is evidence that cellular redox state may affect LXR expression. Cholesterol reacts with ROS and forms products such as oxysterols [243]. Oxysterol levels are increased in NAFLD patients [244], and it is known that they act as potent LXR activators [245]. Therefore, methods to decrease oxidative stress may also reduce expression of LXRs. Nuclear factor erythroid-2-derived like-2 (NFE2L2) is a key transcription factor known to respond to cellular stress by upregulating antioxidant genes. NFE2L2 deficiency results in increased LXR mediated lipogenesis, whereas activation of NFE2L2 by treatment with sulforaphane decreased LXR mediated activation of lipogenic genes [246]. Other compounds such as liquiritigenin have also been shown to inhibit the ability of LXR to induce hepatic steatosis through activation of NFE2L2 [247]. In addition to the beneficial effects of resveratrol discussed earlier, one study demonstrated that resveratrol's ability to decrease lipogenesis may be in part due to decreased LXR expression secondary to upregulation of the antioxidant gene Sestrin2 [97]. Therefore, methods to decrease mitochondrial ROS production may reduce hepatic steatosis by decreased LXR expression.

### 4.5. Farnesoid X Receptor (FXR)

Bile acids play an important role in the absorption of lipids and fat soluble vitamins; however they also possess signaling roles [248]. Bile acids act as ligands for FXR [249], which is critical for lipid and glucose homeostasis as well as cellular inflammatory pathways [250]. FXR promotes fatty acid beta-oxidation and suppresses lipogenesis through its ability to inhibit transcription of SREBP-1c and ChREBP [251, 252]. Indeed, FXR expression has been shown to be decreased in NAFLD patients concomitantly with increased expression of LXR and SREBP-1c [253]. PGC-1*α* has been shown to be an important activator of FXR [254]. This makes physiological sense as increased mitochondrial content would be beneficial for the coupling of the reducing equivalents provided by fatty acid oxidation with oxidative phosphorylation. FXR has not been demonstrated to be directly regulated by oxidative stress; however crosstalk exists between the FXR and LXR pathways. For instance, NFE2L2 activation can deacetylate FXR which can lead to increased levels of small heterodimer partner (SHP) which can inhibit LXR mediated gene transcription [246]. FXR also regulates glucose homeostasis as well, with FXR agonists having been shown to decrease gluconeogenesis through decreased expression of PEPCK, glucose-6-phosphatase, and bisphosphatase while also improving postreceptor insulin signaling [255]. FXR plays an additional role in reducing hepatic inflammation through antagonism of the nuclear factor *κ*B (NF-*κ*B) signaling pathway [256] and reducing the expression of profibrotic genes [257, 258]. FXR agonists such as INT-767 have been tested in models of cholangiopathy with beneficial effects on hepatic inflammation [259]; however they remain to be tested in NAFLD models. The ability of bile acids to activate FXR may explain part of their beneficial effect in NALD. However, bile acids have also been shown to inhibit complex I resulting in a decrease in ATP synthesis similarly to metformin [260].

### 4.6. Pregnane X Receptor (PXR)

PXR has a well-known role in the metabolism and detoxification of a variety of hormones and xenobiotic compounds and has been shown to induce cytochrome P-450 genes necessary for these processes [261]. However, recently PXR has been shown to stimulate lipogenesis by targeting S14, which acts to upregulate the machinery for lipid synthesis [262]. PXR has also been shown to decrease fatty acid oxidation in part due to decreased expression of CPT-1 [263]. Additionally, PXR decreases gluconeogenesis by antagonizing the ability of PGC-1*α* to interact with HNF-4*α* [264] and also by repressing FOXO1, which has been demonstrated to contribute to increased gluconeogenesis in NAFLD [265]. In a mouse model, PXR deletion has been shown to increase mitochondrial fatty acid beta-oxidation, inhibit lipogenesis, decrease inflammation, and improve insulin signaling [266]. Therefore, antagonizing PXR may represent another therapeutic target in NAFLD. Several PXR antagonists have been developed [267, 268] and could potentially be tested for the efficacy in models of NAFLD. However, given PXR's role in drug metabolism its activation or inactivation may significantly alter the pharmacokinetics of other medications. This has been demonstrated for drugs such as rifampicin which activates PXR and has been shown to increase the metabolism of drugs such as antiretroviral medications, oral contraceptives, thiazolidinediones, and benzodiazepines [269]. The converse may be true for PXR antagonists; however detailed studies are still lacking.

### 4.7. SREBP-1c

As discussed above, insulin stimulates fatty acid synthesis largely in part due to activation of SREBP-1c, which activates many steps in the lipogenesis pathway. Even with states of profound insulin resistance, inulin maintains its ability to activate SREBP-1c in NAFLD. In NAFLD, SREBP-1c has been shown to be upregulated in the context of increased expression of insulin receptor substrate- (IRS-)1 and expression of decreased IRS-2 [270]. This decreased expression of IRS-2 relieves inhibition of Forkhead box protein A2 (FoxA2), which has the ability to upregulate fatty acid oxidation [270]. Thus, insulin stimulated SREBP-1c fits well with the concept of increased lipogenesis and high rates of fatty acid beta-oxidation in NAFLD. Increased mitochondrial ROS production likely also contributes to increased SREBP-1c activation in NAFLD. In HepG2 cells, hydrogen peroxide was shown to activate SREB-1c leading to increased lipogenesis [271, 272].

Therefore, some therapeutic approaches have targeted the SREBP-1c signaling pathway. Hydroxytyrosol, which is known to have antioxidant properties, has also been shown to inhibit the SREBP-1c pathway and subsequently prevent liver steatosis and improve insulin resistance in high-fat diet fed C57BL/6J mice [273]. Treatment with hydroxytyrosol also improves expression of mitochondrial subunits and improved mitochondrial ETC enzymatic activity, although it is unclear whether this is due to its inhibition of SREBP-1c or its antioxidant abilities [273]. Herbal medicines have also been shown to inhibit SREBP-1c signaling. Bofutsushosan decreased the expression of SREBP-1c while also affecting a number of other metabolic pathways [274]. This may have been secondary to the ability of this compound to decrease levels of TNF-*α*, which itself has been shown to increase expression of SREBP-1c [275].

Additionally, AMPK activation has also been shown to downregulate SREBP-1c [179, 276], although AMPK expression is not increased in proportion to SREBP-1c [270]. Therefore, targeting the activation of AMPK may also inhibit signaling through SREBP-1c.

## 5. Conclusions

The liver plays a central role in most of the body's metabolic processes, and mitochondria are responsible for performing or coordinating most of these subcellular processes. NAFLD represents impaired regulation of hepatic lipid and glucose homeostasis. While many of the molecular mechanisms involved in the pathogenesis of NAFLD are still being worked out, mitochondria lie at the core of NAFLD. Mitochondria adapt to increasing hepatic lipid content by increasing rates of beta-oxidation and ETC enzymatic activity. However, this leads to the increased production of ROS. This increase in mitochondrial ROS production likely plays an important role in the development of insulin resistance and leads to the function decline in activity of the ETC seen in the later stages of NALD. Mitochondrial functional decline leads to a mismatch between fatty acid beta-oxidation and oxidative phosphorylation leading to the accumulation of partially oxidized intermediates which further exacerbate insulin resistance and leads to the progression of NAFLD. Various interwoven signaling pathways control the cellular metabolic program and are altered to various degrees in NAFLD. Many of these pathways have been shown to be redox sensitive with ROS playing a key modulatory role. Therefore, many antioxidants have been tested in NAFLD with variable results. Other compounds known to modulate upstream signaling pathways have also been tested in NAFLD, many of which have effects on beta-oxidation and other mitochondrial pathways. While several compounds have been tested which are known to upregulate beta-oxidation, without concomitant improvement in the mitochondrial ETC, this approach may further exacerbate insulin resistance and NAFLD. Some studies have reported positive effects of compounds which induce fatty acid beta-oxidation while others report beneficial effects from blocking fatty acid beta-oxidation. The effect of enhancing versus inhibiting fatty acid beta-oxidation may depend on the stage of NAFLD and is also affected by other pathways which are concomitantly modulated. In recent years, much has been learned about the pathogenesis of NAFLD; however few effective and mechanistically targeted therapeutic strategies exist. Part of the difficulty is due to the many overlapping pathways involved in NAFLD and the nonspecific effects of many strategies tested so far. Future treatments will likely need to be developed with greater specificity and their efficacy may vary based on the stage in the pathogenesis of NAFLD.

## Abbreviations

FFA: Free fatty acids
TG: Triglycerides
NASH: Nonalcoholic steatohepatitis
Acetyl-CoA: Acetyl-coenzyme A
ChREBP: Carbohydrate responsive element binding protein
VLDLs: Very low density lipoproteins
CPT-1: Carnitine palmitoyltransferase-1
ETC: Electron transport chain
UCP2: Uncoupling protein 2
ROS: Reactive oxygen species
LFTs: Liver function tests
NAC: N-Acetylcysteine
SNAC: S-Nitroso-N-acetylcsteine
AMPK: AMP activated kinase
SREBP-1c: Sterol regulatory element binding protein 1-c
DGAT2: Diacylglycerol acyltransferase 2
CrAT: Carnitine acetyltransferase
LKB1: Liver kinase B1
CAMKKβ: Calcium calmodulin-dependent protein kinaseβ
ACC: Acetyl-CoA carboxylase
PFK-2: Phosphofructokinase
OCTA1: Organic cation transporter 1
PPAR: Peroxisome proliferator-activated receptor
RXR: Retinoid-X-receptor
TZDs: Thiazolidinediones
TFAM: Mitochondrial transcription factor A
NRF: Nuclear respiratory factor
CREB: cAMP response binding protein
PEPCK: Phosphoenolpyruvate carboxykinase
SOD1: Superoxide dismutase 1
LXR: Liver X receptor
NFE2L2: Nuclear factor erythroid-2-derived like-2
FXR: Farnesoid X receptor
SHP: Small heterodimer partner
NF-*κ*B: Nuclear factor *κ*B
PXR: Pregnane X receptor
IRS: Insulin receptor substrate.
